# Community–based primary health care: a core strategy for achieving sustainable development goals for health

**DOI:** 10.7189/jogh.07.010101

**Published:** 2017-06

**Authors:** Zulfiqar A Bhutta

**Affiliations:** 1 Centre for Global Child Health, The Hospital for Sick Children, Toronto, Canada

A major barrier to the achievement of Millennium Development Goals (MDGs) for reducing maternal and child mortality by three quarters and two–thirds respectively, related to lack of access to care by millions living in abject poverty in rural populations and urban slums [1]. Notwithstanding the challenges, with extraordinary efforts by countries and development partners in the MDG era, child mortality has seen a remarkable decline. Under–five deaths decreased from 12.7 million in 1990 to 5.8 million in 2015 with an increase in the annual rate of under–five mortality reduction from 1.8% over the period 1990–2000 to 3.9% over 2000–2015. However, corresponding progress in reducing neonatal mortality has been less substantial, and the approximate 2.6 million neonates who die every year account for nearly half of all under–five deaths. The annual rate of decrease of neonatal mortality stands at 2.9%, as compared to 4.9% for the 1–59 month age group over this period [2,3].

These gains in global maternal and child health and survival are by no means equal and wide global disparities persist. Sub–Saharan Africa continues to have the highest under–five mortality rate among all regions, with 83 deaths per 1000 live births annually. Although Millennium Development Goal 4 was achieved in some high–mortality countries in Sub–Saharan Africa and South Asia, there are still 58 low– and middle–income countries (LMICs) who are still to reach this target. Barriers to access and equitable care are related to poverty and compounded by lack of trained human resources for health and poorly functional health systems.

In 2015, the world transitioned from the MDGs to the Sustainable Development Goals (SDGs). Although the maternal, neonatal, child and adolescent health issues remain central, the SDGs are all encompassing and the health goal (SDG3) will require close linkages with other contributing SDGs. Although lack of skilled birth attendants and qualified health workers is a large part of the problem, poor health outcomes also related to complex issues such as maternal empowerment, sociocultural taboos, and care–seeking practices and behaviors during pregnancy and child–birth [4]. Given the clustering of maternal and newborn burden of disease in rural settings and among the urban poor we need strategies for promoting community demand as well as appropriate outreach through community health workers and volunteers.

The global evidence base for strategies and interventions for newborn care in community settings has substantially improved, with a range of interventions that can be potentially packaged for delivery at different times during pregnancy, childbirth, and after birth, through various health–care providers, especially community health workers (CHWs). These CHWs were tasked with improving maternal and child health outcomes in a range of research settings and shown to be effective [5]. A fundamental question is whether such concerted interventions especially promotion of behavior–change interventions can be applied in real health systems with busy schedules of health workers and competing priorities. A recent collation of evidence from national programs suggests that this strategy can be effective [6]. Studies evaluating women’s groups, frequently supported by community volunteers show consistent benefits on improved maternal and newborn outcomes [7]. These studies also provide strong evidence that community–based strategies for preventive newborn care substantially improve domiciliary practices, care–seeking patterns, and newborn survival. The case for the scaling–up of strategies for preventive newborn care in community settings with a range of participatory approaches is thus very strong.

Much of the global evidence for community based strategies for care has been collated in systematic reviews with an undue reliance on randomized controlled trials [8]. This supplement collates a vast amount of information from community–based studies of strategies and programs to improve maternal and child health and immunization outcomes, and enhances the evidence in several ways. Dr Perry and colleagues painstakingly collected and reviewed a large body of information from almost 700 reports and studies exploring various aspects of the cumulative experience of community based primary health care globally [9]. The studies comprise of a vast amount of information and while systematic meta–analyses were not performed, the narrative reports demonstrate the effectiveness of various approaches undertaken in disparate geographies and socio–economic settings. These findings are consonant with the findings from a review of community based approaches for primary health care almost a decade ago [10] and the recent evidence synthesis undertaken by the Disease Control Priorities project.

As we move toward accelerating action for achieving the SDGs, the role of community based platforms will become more important. These strategies are needed to ensure that several of the key themes of the SDGs such as reducing inequities and reaching marginalized populations, are achieved within the next 15 years. Some 40 years following the Alma Ata declaration of “health for all”, this might yet become a reality.
